# Supplementary material for: Suppression Head Impulse Test (SHIMP) versus Head Impulse Test (HIMP) When Diagnosing Bilateral Vestibulopathy
Source: J Clin Med. 2022 Apr 26;11(9):2444. doi: 10.3390/jcm11092444 (PMC9102589; doi:10.3390/jcm11092444)
Supplement: Supplementary file 1 [file jcm-11-02444-s001.zip › jcm-1642869-supplementary.pdf]

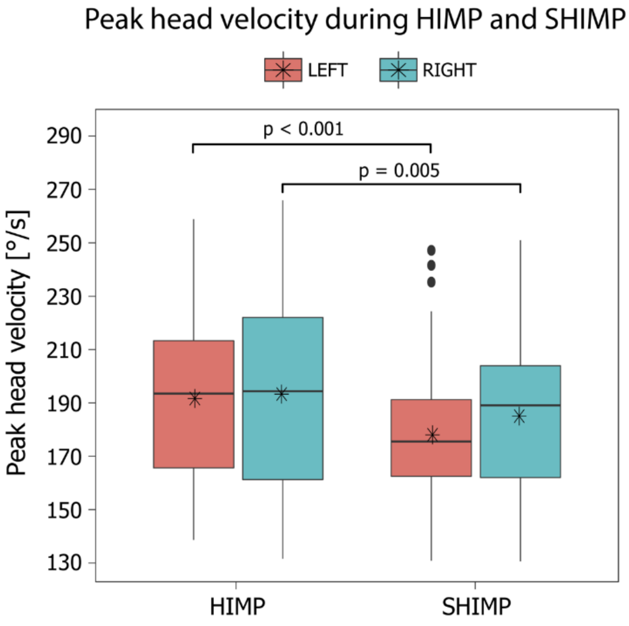

Figure S1. Peak head velocities during HIMP and SHIMP testing in 98 BV patients for rightwards and leftwards head impulses. Black horizontal lines represent median values, asterisks represent mean values for all patients. During SHIMP testing the peak head velocity was statistically significantly lower than during HIMP testing ($p < 0.001$ and $p = 0.005$ for leftwards and rightwards head impulses respectively).
